# Antibacterial Evaluation of Tricyclic Antidepressants Against *S. aureus* and the Possible Pathways of the Mechanism of Action

**DOI:** 10.3390/pathogens14070613

**Published:** 2025-06-20

**Authors:** Vitória Pessoa de Farias Cabral, Daniel Sampaio Rodrigues, Lívia Gurgel do Amaral Valente Sá, Lara Elloyse Almeida Moreira, Cecília Rocha da Silva, João Batista de Andrade Neto, Érica Rayanne Mota da Costa, Thais Lima Ferreira, Leilson Carvalho de Oliveira, Beatriz Oliveira de Souza, Dávylla Rênnia Saldanha Pinheiro, Bruno Coêlho Cavalcanti, Islay Lima Magalhães, Manoel Odorico de Moraes, Hélio Vitoriano Nobre Júnior

**Affiliations:** 1 School of Pharmacy, Laboratory for Bioprospection of Antimicrobial Molecules (LABIMAN), Federal University of Ceará, Fortaleza 60430-372, CE, Brazil; vitoriaffarias15@gmail.com (V.P.d.F.C.); dsampaior@gmail.com (D.S.R.); laraeamoreira@gmail.com (L.E.A.M.); cecilia@ufc.br (C.R.d.S.); joao.neto@unichristus.edu.br (J.B.d.A.N.); ericaraymc@gmail.com (É.R.M.d.C.); thaislimaferreira11@gmail.com (T.L.F.); leilson.ufc@gmail.com (L.C.d.O.); beatrizolivsouza@alu.ufc.br (B.O.d.S.); davyllarennia15@gmail.com (D.R.S.P.); 2 Center for Drug Research and Development (NPDM), Federal University of Ceará, Fortaleza 60430-275, CE, Brazil; bccavalcanti@gmail.com (B.C.C.); islaylm@ufc.br (I.L.M.); odorico@ufc.br (M.O.d.M.); 3 Christus University Center (UNICHRISTUS), Fortaleza 60192-230, CE, Brazil

**Keywords:** *Staphylococcus aureus*, antibacterial, tricyclic antidepressants, amitriptyline, nortriptyline, clomipramine, mechanism of action, synergism

## Abstract

The resistance of *Staphylococcus aureus* to conventional pharmacological treatments has gradually increased. Thus, new therapeutic strategies are needed. Three tricyclic antidepressants (TCAs), amitriptyline (AMT), nortriptyline (NOR), and clomipramine (CLO), stand out with potential in this regard. Thus, the objective of this study was to evaluate the antibacterial activity of TCAs against *S. aureus*. The methodology used broth microdilution, checkerboard, flow cytometry, fluorescence microscopy, and scanning electron microscopy (SEM) techniques. The results showed that the minimum inhibitory concentration (MIC) of AMT was 256 µg/mL, while the MIC of NOR was 128 µg/mL, and the MIC of CLO was between 64 and 128 µg/mL. The TCAs exhibited bactericidal activity. In the analysis of the association with oxacillin (OXA), AMT exhibited 75% synergism, while NOR and CLO obtained 62.5%. In combination with vancomycin (VAN), AMT and NOR presented 100% additive interactions, while CLO exhibited 62.5% indifferent interactions. The mechanism of TCAs, isolated and combined with OXA, was associated with a reduction in cell viability, resulting from their action on the bacterial genetic material and generation of oxidative stress. Furthermore, the action of the drugs produced intense morphological changes in the bacterial cells. In conclusion, TCAs are a potential alternative for antistaphylococcal therapy.

## 1. Introduction

*Staphylococcus aureus* is a commensal bacterium that is often present in sites such as the skin, nostrils, and mucous membranes. However, it has pathogenic potential, being a common cause of human infections worldwide [1]. The clinical manifestations caused by *S. aureus* are varied, ranging from mild skin infections, such as impetigo and abscesses, to chronic wounds and severe diseases like endocarditis and sepsis [2].

Although conventional treatment has been shown to be effective against several *S. aureus* isolates, methicillin-resistant *S. aureus* (MRSA) has a challenging therapeutic approach. Since the emergence of MRSA strains in the United Kingdom in 1961, their incidence has increased [3], becoming a public health concern. MRSA infection leads to increased morbidity and mortality, which, compared to infections caused by methicillin-sensitive *S. aureus* (MSSA), also results in longer hospital stays [4].

The emergence of MRSA associated with its global transmission are important aspects in the epidemiological field [5]. In Ukraine, a multicenter study by Salmanov et al. analyzed the transmission of multidrug-resistant microorganisms and detected resistance to methicillin in 32.4% of the identified *S. aureus* isolates [6]. In a Chinese retrospective study by Yao et al. on the prevalence of MRSA in skin and soft tissue infections, 32.75% of *S. aureus* isolates corresponded to MRSA [7]. In Brazil, a retrospective study by Riche et al. investigated the epidemiology of bloodstream infections caused by *S. aureus* and identified a 22% presence of MRSA strains [8].

According to the World Health Organization, MRSA is a high-priority pathogen to guide research, development, and strategies for the prevention and control of antimicrobial resistance [9]. Drug repositioning is currently used as a means to reduce the time and costs associated with the development of new drugs and can provide new alternatives for the treatment of pathogens resistant to conventionally used antimicrobials. Although originally designed for the treatment of specific diseases, many drugs have secondary antimicrobial properties. Furthermore, the combination of repositioned drugs with existing antibiotics has the potential to increase antibacterial action through synergistic interactions [10].

In this context, several antidepressants have been reported to have antimicrobial properties [11], demonstrating the ability to disrupt the integrity of the bacterial cytoplasmic membrane and thereby compromise its function as a selective permeability barrier [12]. The class of tricyclic antidepressants (TCAs), comprising amitriptyline (AMT), nortriptyline (NOR), and clomipramine (CLO) [13], classically act by inhibiting the reuptake of serotonin and norepinephrine, resulting in the accumulation of these neurotransmitters in the presynaptic cleft. Most TCAs have been approved by the US Food and Drug Administration (FDA) for the treatment of depression and anxiety disorders and are also considered viable options for off-label use, such as the treatment of pain syndromes [14].

In the literature, the antibacterial activity of TCAs is described mainly with AMT, with fewer studies focusing on NOR and CLO [15,16,17,18,19,20]. Additionally, there is a lack of studies evaluating the action of these TCAs associated with antibacterials against clinical isolates of MSSA and MRSA. Furthermore, the mechanisms by which these antidepressants act as antibacterials have not been elucidated. Therefore, the objective of this study was to evaluate the antibacterial activity of AMT, NOR, and CLO against clinical strains of MSSA and MRSA, both alone and associated with OXA and VAN, as well as to analyze the possible pathways of TCAs’ antistaphylococcal action.

## 2. Materials and Methods

### 2.1. Bacterial Isolates and Drugs

Seven clinical isolates of *S. aureus* were used, four of which were MRSA and three of which were MSSA. In addition, the *S. aureus* ATCC 6538p strain was used as a control. All isolates belong to the microorganism collection of the Laboratory for Bioprospection of Antimicrobial Molecules (LABIMAN) of Federal University of Ceará (UFC).

The TCAs (AMT, NOR, and CLO) were purchased from Fagron (São Paulo, Brazil) and were solubilized in sterile distilled water. In turn, the two antibacterials, oxacillin (OXA) and vancomycin (VAN), were purchased from Sigma-Aldrich (St. Louis, MO, USA) and were also dissolved in sterile distilled water.

### 2.2. Antimicrobial Susceptibility Testing (AST) and Breakpoints

AST was performed according to Clinical and Laboratory Standards Institute (CLSI) document M07-A10 [21]. TCAs were evaluated in the concentration range of 1024–2 µg/mL. OXA was tested in the range of 1024–2 µg/mL for MRSA strains, and in the range of 2–0.004 µg/mL for MSSA. VAN was tested in the range of 64–0.125 µg/mL. The minimum inhibitory concentration (MIC) was established as the lowest concentration capable of visually completely inhibiting bacterial growth after the incubation period.

For the antibacterials, breakpoints were analyzed according to CLSI document M100-S31 [22]. For OXA, isolates with an MIC ≤ 2 µg/mL were classified as MSSA, and MIC ≥ 4 µg/mL as MRSA. For VAN, strains were classified as susceptible (≤2 µg/mL) according to this same document.

### 2.3. Assessment of Minimum Bactericidal Concentration (MBC) and Tolerance Level

After reading the AST, aliquots (10 µL) were taken from the wells corresponding to the MIC, 2× MIC, and 4× MIC. These were plated on brain heart infusion (BHI) agar (HiMedia, Mumbai, MH, India), and the plates were incubated for 24 h at 35 °C. After this period, the lowest concentration at which no growth was observed on the agar surface was recorded as the MBC [23].

From this, a ratio between the MBC/MIC was calculated to obtain the tolerance level. For bactericidal action, the resulting ratio was ≤4, and for bacteriostatic action, the ratio corresponded to >4 [24].

### 2.4. Analysis of the Interaction Between Antibacterials and TCAs

The associated action of OXA and VAN with TCAs was evaluated using the checkerboard technique [25]. From the MICs of the isolated drugs, single solutions of the drugs were prepared for each isolate and combination. From each solution, a serial twofold dilution was performed. Thus, the associated MICs were analyzed according to the fractional inhibitory concentration index (FICI), corresponding to the ratio between the MIC_ASSOCIATED(TCA)_/MIC_ISOLATED(TCA)_ and MIC_ASSOCIATED(ANTIBACTERIAL)_/MIC_ISOLATED(ANTIBACTERIAL)_. The results were interpreted according to Jorge et al. [26] as synergistic (FICI ≤ 0.5), additive (0.5 < FICI ≤ 1), indifferent (1 < FICI ≤ 4), or antagonistic (FICI > 4).

### 2.5. Possible Mechanism of Action

#### 2.5.1. Treatment of Cells

For these assays, the representative MRSA isolate 1 was used. Initially, the cells were collected during exponential growth in BHI broth medium (HiMedia, Mumbai, MH, India). From these, an inoculum containing 10^6^ cells/mL was prepared. The cells were incubated with the drugs for 24 h at 35 °C [23]. Thus, the treatments consisted of the isolated and associated MIC of TCAs with oxacillin for the MRSA isolate 1, which corresponded to the following: AMT (256 µg/mL), NOR (128 µg/mL), CLO (64 µg/mL), AMT + OXA (AMT 64 µg/mL + OXA 16 µg/mL), NOR + OXA (NOR 32 µg/mL + OXA 16 µg/mL), CLO + OXA (CLO 16 µg/mL + OXA 16 µg/mL). The associated evaluation of TCAs and OXA was performed in view of the promising results obtained in the checkerboard assay. Furthermore, treatment with OXA was also performed at the MIC (64 µg/mL) and at the subinhibitory concentration (16 µg/mL) present in the combination (OXA SUB), performed for comparison purposes. VAN (5 µg/mL) was additionally used as a death control.

#### 2.5.2. Determination of Cell Viability

The analysis was performed with the exclusion assay using propidium iodide (PI) (2 µg/mL). Aliquots of the samples were collected for the evaluation of cellular fluorescence using a FACSCalibur cytometer (Becton Dickinson, San Jose, CA, USA). Ten-thousand events were evaluated per experiment, with cellular debris being omitted [27,28,29].

#### 2.5.3. Assessment of DNA Fragmentation

The terminal deoxynucleotidyl transferase dUTP nick-end labeling (TUNEL) assay was used to verify DNA fragmentation from the treatments performed. The test was performed following the recommendations of the kit’s manufacturer (Roche, Rotkreuz, Switzerland). Thus, the cells were fixed with 7% paraformaldehyde and added to 1% Triton X-100 for 10 min on ice. Then, they were incubated for 1 h at 37 °C with the TUNEL mixture [30,31]. Two-hundred cells were counted to determine the percentage of TUNEL-positive cells and examined using a fluorescence microscope (Olympus, Tokyo, Japan).

#### 2.5.4. Alkaline Comet Test

The analysis was performed according to Dong et al. [32] by single-cell gel electrophoresis. Cells were collected and combined with agarose at a ratio of 1:10 (*v*/*v*) and placed on glass slides that were kept at a temperature of 4 °C in the dark for 15 min. The slides were then transferred to 25 mL of RIPA lysis buffer for 30–60 min at 4 °C in the dark, followed by induction using an alkaline solution for 30 min at 4 °C and washing with pre-cooled tris-boric acid electrophoresis buffer. The glass slides were rinsed with 70% ethanol for 5 min and air-dried. Vista Green DNA dye was added to the bacterial samples, and these were observed using a fluorescence microscope (Olympus, Tokyo, Japan).

#### 2.5.5. Quantification of Reactive Oxygen Species (ROS)

Detection was performed by incubating bacterial cells in 20 µM CM-H_2_DCFDA [5-(and-6)-chloromethyl-2′,7′-dichlorodihydrofluorescein diacetate acetyl ester] (Sigma-Aldrich, St. Louis, MO, USA) for 30 min at 35 °C in the absence of light. After the cells were collected, they were washed and resuspended in sodium phosphate buffer (PBS) and analyzed by flow cytometry (FACSCalibur, Becton Dickinson, San Jose, CA, USA) [33,34].

#### 2.5.6. Analysis of Carbonyl Proteins

After treatments, cells were lysed, the supernatant was collected, and two 100 µL aliquots of samples were taken, which received 300 µL of 2,4-dinitrophenylhydrazine (DNPH) and 300 µL of 2.5 M HCl, respectively, and were incubated for 1 h at room temperature with shaking. After this period, 500 µL of 20% trichloroacetic acid was added to each sample, with vortexing and cold incubation. After 5 min, the resulting sample pellets were resuspended in 500 µL of 10% trichloroacetic acid. Then, the sediment was resuspended in 500 µL of ethanol/ethyl acetate (1:1, *v*/*v*) using vortexing, and the pellets were resuspended in 250 µL of guanidine hydrochloride, in which the supernatant was used for reading [35]. The reading was performed spectrophotometrically (SpectraCount, Packard, Mississauga, ON, Canada) at 370 nm. The total carbonylation content of the protein was determined as nmol/mg of protein.

### 2.6. Data Analysis

The experiments were performed on different days and with independent replicates, each performed in triplicate. The data regarding the mechanism of action were submitted to a one-way analysis of variance (ANOVA), followed by the Tukey test, in which *p* ˂ 0.05 was considered significant. The analyses were performed using the GraphPad Prism program (version 8 for Windows, GraphPad Software, San Diego, CA, USA).

### 2.7. Scanning Electron Microscopy (SEM)

SEM was performed with the same representative MRSA isolate 1. Briefly, an inoculum (5 × 10^5^ CFU/mL) was prepared in tryptone soy broth (TSB) (Kasvi, Pinhais, PR, Brazil) supplemented with 2% glucose (Isofar, Rio de Janeiro, Brazil). Treatments with TCAs alone and in combination with OXA, as well as with OXA at MIC and OXA SUB, were performed in this inoculum, with incubation for 24 h at 35 °C. Treated cells were added to coverslips pretreated with 2.5% silane and fixed with a solution containing glutaraldehyde (2.5%) and sodium cacodylate buffer (0.15 M). Immediately afterwards, they were incubated at 4 °C overnight. After this period, the coverslips were washed with cacodylate buffer solution (0.15 M) and dehydrated with increasing concentrations of ethyl alcohol (Dinâmica, São Paulo, Brazil). After drying, they were covered with hexamethyldisilazane (Sigma-Aldrich, St. Louis, MO, USA) at room temperature, until the reagent was completely dry [23]. The coverslips were coated with a gold layer (Emitech, Uckfield, UK; Q150T) (10 nm) and visualized under an FEI Quanta 450 FEG microscope (FEI Company, Waltham, MA, USA).

## 3. Results

### 3.1. TCAs Exhibited Antibacterial Activity Against S. aureus and Had a Bactericidal Action Profile

The MSSA isolates presented MICs ranging from 0.125 to 1 µg/mL for OXA, and the MRSA isolates presented MICs of 64 µg/mL for this same drug. Regarding VAN, the isolates obtained MICs between 1 and 2 µg/mL. With regard to the potential of TCAs against MSSA and MRSA, AMT exhibited inhibitory action at 256 µg/mL, NOR at 128 µg/mL, and CLO between 64 and 128 µg/mL (Table 1).

In addition, the three TCAs exhibited a bactericidal action profile. AMT presented an MBC of 256 µg/mL, whereas for NOR, this was obtained in the range from 128 to 256 µg/mL, and for CLO between 64 and 128 µg/mL. The tolerance level for AMT corresponded to 1, and for NOR and CLO, this metric remained between 1 and 2 (Table 1).

### 3.2. The Association Between TCAs and OXA Demonstrated a Synergistic Effect Against S. aureus

In the analysis of the associated activity, a predominance of synergistic interactions between TCAs and OXA was observed. Thus, AMT associated with OXA exhibited 75% synergism, and NOR and CLO, in combination with this antibacterial, obtained 62.5% synergistic interactions. In the association between TCAs and VAN, both AMT and NOR presented 100% additive interactions. For CLO, 62.5% of the interactions were indifferent in the combination with VAN (Table 2).

### 3.3. TCAs Reduced MRSA Viability

**Figure 1:**
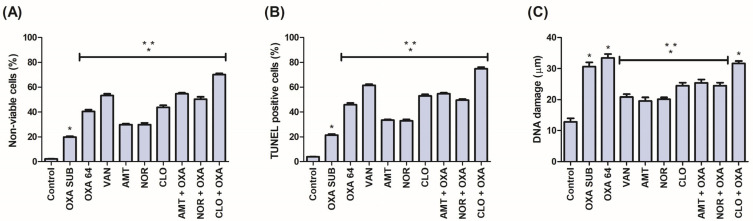
(**A**) Cell viability of MRSA cells treated with TCAs, assessed by the PI assay. (**B**) DNA fragmentation of MRSA cells treated with TCAs, evaluated using the TUNEL assay. (**C**) DNA damage of MRSA cells treated with TCAs, assessed by the comet assay. Significance was defined as * *p* < 0.05 compared with the respective control and ** *p* < 0.05 compared with OXA SUB by the Tukey test. The control refers to untreated cells. All tests were performed in at least three replicates.

The treatment of MRSA cells with TCAs alone and in combination with OXA reduced the number of viable cells (Figure 1A). Statistical significance (*p* < 0.05) was observed for an increase in the percentage of nonviable cells in comparison with the control (2.11 ± 0.65%) and with OXA SUB (19.86 ± 2.28%). Therefore, the action of AMT resulted in 29.76 ± 2.48% of nonviable cells, similar to NOR, which obtained 29.72 ± 4.46%. CLO generated a stronger effect, with 43.76 ± 5.28%. In combination with OXA, AMT generated 54.62 ± 2.47% of nonviable cells, also similar to NOR, with 50.26 ± 5.97%. CLO also stood out in the association with OXA, generating 70.30 ± 2.76%. VAN (death control) obtained 53.19 ± 4.34%, and OXA 64 at 40.55 ± 4.21%.

### 3.4. Treatment of MRSA with TCAs Resulted in an Increase in TUNEL-Positive Cells

The action of TCAs alone and in association with OXA induced DNA fragmentation, as evidenced by the TUNEL assay results (Figure 1B), a marker of cell death. Statistical significance (*p* < 0.05) was verified in the comparison with the control (3.85 ± 0.48%) and OXA SUB (21.56 ± 2.35%). TUNEL-positive cells corresponded to 33.44 ± 2.05% for AMT, 32.94 ± 3.20% for NOR, 52.90 ± 4.23% for CLO, 54.62 ± 2.47% for AMT + OXA, 49.61 ± 2.36% for NOR + OXA, and 74.98 ± 3.87% for CLO + OXA. The VAN death control exhibited 61.51 ± 3.18% TUNEL-positive cells, and OXA 64 exhibited 45.98 ± 4.17%.

### 3.5. The Action of TCAs on DNA Evidenced by the Alkaline Comet Assay

DNA damage produced by the treatment of MRSA cells with TCAs was confirmed using the alkaline comet assay, a test that detects both direct and indirect DNA lesions. TCAs alone and in combination with OXA generated significant (*p* < 0.05) DNA damage (Figure 1C) compared to the control (12.81 ± 3.43 µm), also verified in OXA SUB (30.69 ± 3.94 µm) and OXA 64 (33.37 ± 3.83 µm). AMT (19.54 ± 3.58 µm), NOR (20.15 ± 1.73 µm), CLO (24.48 ± 2.99 µm), and VAN (20.85 ± 2.70 µm) exhibited similar isolated profiles, and in the associations between AMT + OXA (25.41 ± 3.25 µm), NOR + OXA (24.50 ± 2.97 µm), and CLO + OXA (31.72 ± 2.03 µm), greater damage was generated by CLO.

### 3.6. ROS Production Occurred in the Action of TCAs Against MRSA

**Figure 2:**
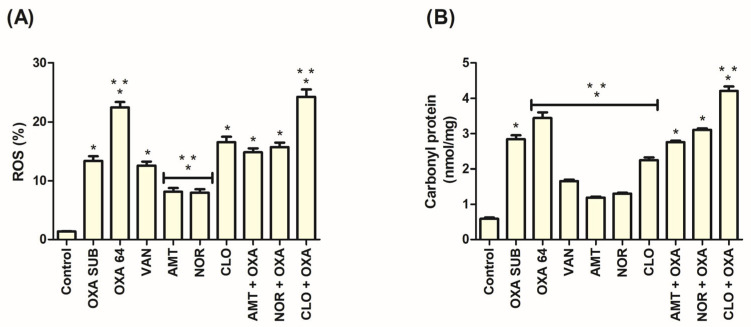
(**A**) ROS levels in MRSA cells treated with TCAs, assessed using CM-H_2_DCFDA. (**B**) Protein carbonylation in MRSA cells treated with TCAs. Significance was defined as * *p*  <  0.05 compared with the respective control and ** *p*  <  0.05 compared with OXA SUB by the Tukey test. The control refers to untreated cells. All tests were performed in at least three replicates.

The ROS level in the treatment of MRSA cells with TCAs was significantly (*p* < 0.05) increased compared to the control (1.37 ± 0.16%) (Figure 2A). For TCAs alone, this metric corresponded to 8.13 ± 1.54% for AMT, 7.95 ± 1.47% for NOR, and 16.55 ± 2.24% for CLO. In the combinations, the results were 14.82 ± 1.61% for AMT + OXA, 15.71 ± 1.84% for NOR + OXA, and 24.28 ± 2.95% for CLO + OXA. A significant increase in comparison with OXA SUB (13.36 ± 1.98%) was observed only in the combination CLO + OXA among the TCAs. OXA 64 obtained 22.50 ± 2.22% and VAN obtained 12.55 ± 1.66%.

### 3.7. Increased Protein Carbonylation Was Associated with the Action of TCAs in MRSA

In the analysis of the irreversible protein damage marker, TCAs achieved significance (*p* < 0.05) in comparison with the control (0.58 ± 0.09 nmol/mg) (Figure 2B). Treatment with AMT resulted in 1.19 ± 0.07 nmol/mg, NOR in 1.30 ± 0.08 nmol/mg, CLO in 2.25 ± 0.19 nmol/mg, AMT + OXA in 2.75 ± 0.10 nmol/mg, NOR + OXA in 3.11 ± 0.11 nmol/mg, CLO + OXA in 4.20 ± 0.31 nmol/mg, OXA SUB in 2.84 ± 0.27 nmol/mg, OXA 64 in 3.44 ± 0.35 nmol/mg, and VAN in 1.66 ± 0.10 nmol/mg. Among the TCAs, only the CLO + OXA combination resulted in a significant increase compared to OXA SUB alone.

### 3.8. The Antibacterial Activity of TCAs Generated Considerable Morphological Changes in MRSA Bacterial Cells

**Figure 3:**
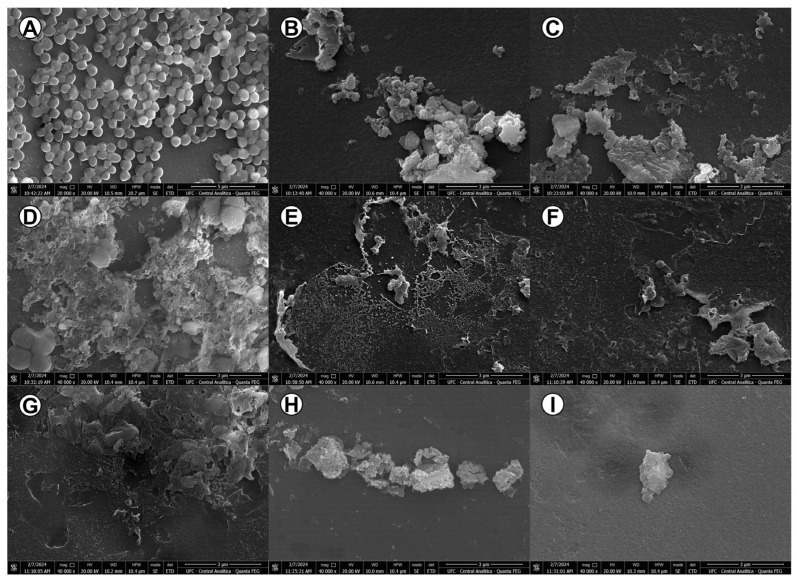
Morphology of MRSA planktonic cells treated with TCAs isolated and associated with OXA by SEM. (**A**): Control, B-I: treatments with AMT (**B**), NOR (**C**), CLO (**D**), OXA (**E**), OXA SUB (**F**), AMT + OXA (**G**), NOR + OXA (**H**), and CLO + OXA (**I**). Magnification (**A**): 20,000×, bar: 5 µm; magnification (**B**–**I**): 40,000×, bar: 3 µm.

Figure 3A represents the control used in the SEM, demonstrating cocci grouped in clusters, characteristic of the *Staphylococcus* genus, with intact bacterial cells. The treatments with AMT, NOR, and CLO (Figure 3B, Figure 3C, and Figure 3D, respectively) caused intense microbial destruction, producing cell debris and a loss of conventional morphology. The treatments with OXA at MIC (Figure 3E) and OXA SUB (Figure 3F) also resulted in extensive morphological damage. In the combined evaluation, AMT + OXA (Figure 3G), NOR + OXA (Figure 3H), and CLO + OXA (Figure 3I) generated the same pattern exhibited by the isolated drugs, evidencing changes in microbial integrity.

## 4. Discussion

The microorganisms classified in the ESKAPE group, which includes *S. aureus*, are recognized for sharing specific characteristics, such as their ability to survive in healthcare environments and their repertoire of intrinsic or acquired resistance determinants, making them a relevant emerging cause of resistant infections [36].

The rapid evolution of bacteria poses significant challenges to the development of new antimicrobials, often rendering them economically inefficient. Consequently, drug repurposing has emerged as an alternative strategy, recognized as having promising potential against *S. aureus* [37].

AMT was reported in a previous study by Machado et al. [15] to have antibacterial activity, with a 100% MIC of 128 µg/mL against the *S. aureus* ATCC 29,213 strain and a MIC of 512 µg/mL versus *S. aureus* ATCC 25923, values close to those found in our study. They also observed a bactericidal action profile. In studies by Kalayci, Demirci and Sahin [19], and Munoz-Bellido, Munoz-Criado, and Gracìa-Rodrìguez [18], MICs of 100% and 90%, respectively, corresponding to 32 µg/mL and 128 µg/mL, were observed for CLO against *S. aureus* isolates, similar results to ours.

Regarding NOR, there is a lack of studies evaluating its antibacterial potential. Rajamohan, Subramania, and Lee [38] studied the application of this TCA through a nanocarrier, but few promising results were observed in the scope of antimicrobial action, so more research is needed.

In the present study, we found that the MICs of AMT, NOR, and CLO mostly followed a descending order. These data also corroborate the findings of Cabral et al. [39] regarding the action of TCAs against *Candida* spp., in which the MIC 50% values were in the range from 16 to 128 µg/mL for AMT, 8 to 128 µg/mL for NOR, and 8 to 64 µg/mL for CLO. According to these authors, this variation in activity possibly occurred due to the structure–activity relationship, considering the presence of the chlorine element in CLO, and the difference in the methyl group between AMT and NOR.

With respect to the associated activity between TCAs and antibacterials, Machado et al. [15] combined AMT with ciprofloxacin, sulfamethoxazole-trimethoprim, and colistin, identifying 15 synergistic combinations against Gram-positive and Gram-negative bacteria, as well as other types of interactions among the evaluated isolates.

Ugurel and Turgut [17] also evaluated AMT associated with gentamicin and tetracycline against *Acinetobacter baumannii*, observing indifferent and additive interactions, respectively. Otto et al. [20] evaluated AMT combined with polymyxin B against Gram-negative bacteria, finding synergism in all isolates of *A. baumannii* and *Escherichia coli* evaluated, and in three isolates of *Klebsiella pneumoniae*.

Another important aspect was addressed by Peng et al. [40]. The authors found that in mice infected with *S. aureus*, which were treated with a combination of antibiotics (methicillin or VAN) and AMT, there was a reduction in pulmonary edema and bacteremia, thus protecting the rats from lethal sepsis and pulmonary dysfunction. This combination of effects was not observed in treatments with the drugs alone, highlighting the relevance of the possible association of AMT and other TCAs with antibiotics.

In combination with OXA, the interactions observed with TCAs were mostly synergistic, unlike what was found with the association of VAN, for which most interactions were additive with AMT and NOR, and indifferent with CLO. Considering the greater potential shown by the combination with OXA, the analysis of the possible mechanisms involved in the antibacterial action was also performed regarding the association with this antibiotic.

It is noteworthy that β-lactam antibiotics act by interrupting peptidoglycan synthesis and inactivating penicillin-binding proteins (PBPs), interfering with the assembly of the bacterial cell wall, which protects the plasma membrane from osmotic rupture [41]. TCAs are known to alter the structural organization of lipid membranes [42]. Hence, the synergism obtained between TCAs and OXA is possibly due to a potentiation of the effect between the drugs obtained at the structural level, as visualized by SEM. Since glycopeptides inhibit a different stage of bacterial cell wall synthesis [43], different interactions with TCAs were observed, resulting in less intensification of the effect of combined VAN compared to OXA. The different PBP targets or cell wall-binding sites may have influenced the observed interactions with the antibacterials. Further studies should be carried out to determine the variants associated with these interactions.

Based on the results of the present study, the possible pathways of action of TCAs alone and in association with OXA against *S. aureus* involve a reduction in cell viability resulting from the action of these drugs on bacterial genetic material and the generation of oxidative stress. Furthermore, the action of the drugs caused intense morphological changes in bacterial cells, visualized by SEM, corroborating the potent bactericidal action.

Machado et al. [15] found that AMT was able to cleave plasmid DNA at 3.75 and 1.875 mM at pH 7.4 at 50 °C, which did not occur at lower concentrations (0.375 mM), as well as at 37 °C, where it caused DNA damage. Considering that bacterial plasmids are extrachromosomal elements that can carry genes that confer antibiotic resistance, the antiplasmid effect of TCAs opens perspectives for dealing with the main problem of multidrug-resistant bacterial strains [44]. Further research should also be carried out to evaluate the antiplasmid effect of TCAs under ideal growth conditions for *S. aureus*. In the present study, significant correlations were observed in the tests concerning the action of TCAs on DNA.

In a study by Dwyer et al. [31], the authors reported that bactericidal antibiotics, such as ampicillin and a β-lactam, induced detectable DNA fragmentation by the TUNEL assay, a test that has been used as an indicator of cell death. This finding is consistent with the data for OXA, which belongs to the same pharmacological class, where the effect was enhanced by the combination with TCAs. In the comet assay, an approach developed to quantify DNA damage, it has been observed that the more severe the DNA damage is, the farther it migrates under electrophoretic conditions [32], identifying both direct and indirect DNA lesions, which have also been shown to be associated with the action mechanism of TCAs.

Furthermore, the increased production of ROS and carbonyl proteins suggests that the antistaphylococcal mechanism of TCAs is associated with oxidative stress. In the literature, there are no reports about this parameter involving the antibacterial action of TCAs, so there is a lack of data to elucidate the activity of these antidepressants against prokaryotic microorganisms. However, a study by Cabral et al. [39] found that ROS generation and protein carbonylation was associated with the anti-*Candida* mechanism of TCAs. In addition, studies by Da Silva Rodrigues et al. [45,46] found there was an increase in ROS production in the antiparasitic action of CLO against *Trypanosoma brucei* and *Leishmania amazonensis*.

The development of treatments based on the generation of oxidative stress, aiming at the redox imbalance of bacterial pathogens, is a relevant strategy that has received increased attention in recent years. This approach allows the identification of antimicrobials with repurposing potential that can be part of combination therapies, including against infections caused by recalcitrant bacterial pathogens [47]. For future studies, it is suggested to investigate the possibility that ROS generation may also occur as a secondary effect resulting from membrane damage. Furthermore, experiments using ROS scavengers may help to further elucidate the involvement of oxidative stress in this mechanism.

Since *S. aureus* is a common skin-associated pathogen, repositioned drugs with antibacterial potential are considered as alternatives for topical use, either as single agents or combined with conventional antimicrobials, in order to increase the efficacy and extend the shelf life of these substances [48]. The use of antidepressants in dermatology is reported in the literature based on other pharmacological properties not directly related to the antidepressant effect [49]. Thus, the use of TCAs is considered a potential strategy for the treatment of infections caused by *S. aureus*.

For skin applications, the use of topical formulations is proposed. Previous studies have demonstrated the use of TCAs in pharmaceutical forms via this route [50,51,52,53], including at high concentrations. Therefore, future studies are needed to evaluate the potential applications of these TCAs in topical formulations and in vivo models.

The present study brings new observations about the antibacterial activity of TCAs against *S. aureus*, elucidating their interactions with antibacterials relevant for the treatment of infections caused by this bacterium, and clarifying possible mechanisms associated with their antimicrobial action, aspects with scarce data in the literature.

## 5. Conclusions

TCAs had antibacterial activity against MSSA and MRSA, exhibiting a bactericidal action profile and mostly synergistic interactions in association with OXA. The action pathways of TCAs, isolated and combined with OXA, were associated with a reduction in cell viability resulting from their action on genetic material and production of oxidative stress. Treatment with the drugs generated intense morphological changes in bacterial cells, corroborating their potent bactericidal action. In summary, TCAs are characterized as promising alternatives for antistaphylococcal therapy.

## Figures and Tables

**Table 1:** Antibacterial effects of TCAs against *S. aureus* strains.

Strains ^a^	OXA ^b^	VAN ^c^	AMT ^d^				NOR ^e^				CLO ^f^			
	MIC ^g^	MIC ^g^	MIC ^g^	MBC ^h^	MBC ^h^/MIC ^g^	Interpretation	MIC ^g^	MBC ^h^	MBC ^h^/MIC ^g^	Interpretation	MIC ^g^	MBC ^h^	MBC ^h^/MIC ^g^	Interpretation
MSSA ATCC 6538p	0.125	1	256	256	1	Bactericide	128	256	2	Bactericide	64	64	1	Bactericide
MSSA 1	0.5	1	256	256	1	Bactericide	128	128	1	Bactericide	64	64	1	Bactericide
MSSA 2	1	1	256	256	1	Bactericide	128	128	1	Bactericide	64	128	2	Bactericide
MSSA 3	1	1	256	256	1	Bactericide	128	256	2	Bactericide	64	64	1	Bactericide
MRSA 1	64	1	256	256	1	Bactericide	128	256	2	Bactericide	64	64	1	Bactericide
MRSA 2	64	2	256	256	1	Bactericide	128	256	2	Bactericide	128	128	1	Bactericide
MRSA 3	64	2	256	256	1	Bactericide	128	128	1	Bactericide	64	64	1	Bactericide
MRSA 4	64	1	256	256	1	Bactericide	128	256	2	Bactericide	64	64	1	Bactericide

^a^ *S. aureus* strains belonging to the arsenal of isolates of the Laboratory of Bioprospection of Antimicrobial Molecules (LABIMAN). ^b^ OXA—oxacillin. ^c^ VAN—vancomycin. ^d^ AMT—amitriptyline. ^e^ NOR—nortriptyline. ^f^ CLO—clomipramine. ^g^ MIC (minimum inhibitory concentration) was defined as the lowest concentration capable of inhibiting the growth of bacterial cells in 100% after incubation. Broth microdilution was performed according to the CLSI protocol (M07-A10). ^h^ MBC—minimum bactericidal concentration. The mean was used for data analysis.

**Table 2:** Associated activity between TCAs and antibacterials against *S. aureus* strains.

Strains ^a^	MIC_100%_ Combination ^b^ (µg/mL)																	
	AMT ^c^/OXA ^d^	FICI ^e^	INT ^f^	AMT ^c^/VAN ^g^	FICI ^e^	INT ^f^	NOR ^h^/OXA ^d^	FICI ^e^	INT ^f^	NOR ^h^/VAN ^g^	FICI ^e^	INT ^f^	CLO ^i^/OXA ^d^	FICI ^e^	INT ^f^	CLO ^i^/VAN ^g^	FICI ^e^	INT ^f^
MSSA ATCC 6538p	128/0.0625	1	ADI	128/0.5	1	ADI	64/0.0625	1	ADI	64/0.5	1	ADI	64/0.125	2	IND	32/0.5	1	ADI
MSSA 1	128/0.25	1	ADI	128/0.5	1	ADI	64/0.25	1	ADI	64/0.5	1	ADI	32/0.25	1	ADI	64/1	2	IND
MSSA 2	64/0.25	0.5	SYN	128/0.5	1	ADI	32/0.25	0.5	SYN	64/0.5	1	ADI	32/0.5	1	ADI	64/1	2	IND
MSSA 3	64/0.25	0.5	SYN	128/0.5	1	ADI	64/0.5	1	ADI	64/0.5	1	ADI	16/0.25	0.5	SYN	64/1	2	IND
MRSA 1	64/16	0.5	SYN	128/0.5	1	ADI	32/16	0.5	SYN	64/0.5	1	ADI	16/16	0.5	SYN	64/1	2	IND
MRSA 2	64/16	0.5	SYN	128/1	1	ADI	32/16	0.5	SYN	64/1	1	ADI	32/16	0.5	SYN	64/1	1	ADI
MRSA 3	32/8	0.25	SYN	128/1	1	ADI	32/16	0.5	SYN	64/1	1	ADI	16/16	0.5	SYN	32/1	1	ADI
MRSA 4	32/8	0.25	SYN	128/0.5	1	ADI	32/16	0.5	SYN	64/0.5	1	ADI	16/16	0.5	SYN	64/1	2	IND

^a^ *S. aureus* strains belonging to the arsenal of isolates of the Laboratory of Bioprospection of Antimicrobial Molecules (LABIMAN).^b^ MIC_100%_ of TCAs associated with antibacterials and its interactions. ^c^ AMT—amitriptyline. ^d^ OXA—oxacillin. ^e^ FICI—fractional inhibitory concentration index. ^f^ INT—interaction. ^g^ VAN—vancomycin. ^h^ NOR—nortriptyline. ^i^ CLO—clomipramine. The mean was used for data analysis.

## Data Availability

The original contributions presented in this study are included in the article. Further inquiries can be directed to the corresponding authors.

## Abbreviations

The following abbreviations are used in this manuscript:

ADI	Additive
AMT	Amitriptyline
ANOVA	Analysis of variance
AST	Antimicrobial susceptibility testing
BHI	Brain heart infusion
CFU	Colony-Forming Units
CLSI	Clinical and Laboratory Standards Institute
CLO	Clomipramine
DNA	Deoxyribonucleic acid
DNPH	2,4-dinitrophenylhydrazine
ESKAPE	*Enterococcus faecium*, *Staphylococcus aureus*, *Klebsiella pneumoniae*, *Acinetobacter baumannii*, *Pseudomonas aeruginosa*, and *Enterobacter* spp.
FDA	Food and Drug Administration
FICI	Fractional Inhibitory Concentration Index
IND	Indifferent
LABIMAN	Laboratory for Bioprospection of Antimicrobial Molecules
MBC	Minimum Bactericidal Concentration
MBC/MIC	Tolerance level
MIC	Minimum Inhibitory Concentration
MRSA	Methicillin-resistant *S. aureus*
MSSA	Methicillin-sensitive *S. aureus*
NOR	Nortriptyline
OXA	Oxacillin
OXA SUB	Subinhibitory oxacillin
PI	Propidium iodide
ROS	Reactive oxygen species
SEM	Scanning electron microscopy
SYN	Synergism
TCAs	Tricyclic antidepressants
TUNEL	Terminal deoxynucleotidyl transferase dUTP nick-end labeling
UFC	Federal University of Ceará
VAN	Vancomycin
